# Novel Gluten-Free Bread with an Extract from Flaxseed By-Product: The Relationship between Water Replacement Level and Nutritional Value, Antioxidant Properties, and Sensory Quality

**DOI:** 10.3390/molecules27092690

**Published:** 2022-04-21

**Authors:** Urszula Krupa-Kozak, Natalia Bączek, Vanessa D. Capriles, Łukasz Łopusiewicz

**Affiliations:** 1 Institute of Animal Reproduction and Food Research of Polish Academy of Sciences, Tuwima 10 Street, 10-748 Olsztyn, Poland; n.baczek@pan.olsztyn.pl; 2 Laboratory of Food Technology and Nutrition, Department of Biosciences, Institute of Health and Society, Federal University of São Paulo, Santos 11015-020, Brazil; vanessa.capriles@unifesp.br; 3 Center of Bioimmobilisation and Innovative Packaging Materials, Faculty of Food Sciences and Fisheries, West Pomeranian University of Technology, Klemensa Janickiego 35 Street, 71-270 Szczecin, Poland

**Keywords:** co-product revalorization, *Linum usitatissimum* L., flaxseed oil, post-production waste, gluten-free products, breadmaking, bread quality, QDA, bioactive potential

## Abstract

The food industry generates a great amount of food waste and by-products, which in many cases are not fully valorized. Press cakes, deriving from oilseeds extraction, represent interesting co-products due to their nutritional value, high biopolymers content, and the presence of bioactive phytochemicals. Gluten-free breads (GFBs) are products that have disadvantages such as unsatisfactory texture, low nutritional value, and short shelf life, so natural additives containing proteins and hydrocolloids are in demand to increase GFBs value. In this study, extract from flaxseed by-product (FOCE—Flaxseed Oil Cake Extract) was used to replace water (25–100%) in GFBs formulations and their nutritional value, antioxidant properties, and sensory features were investigated. The results showed that GFBs with FOCE had an elevated nutritional and nutraceutical profile (up to 60% more proteins, significantly increased K, Mg, and P levels). Moreover, the addition of FOCE improved the technological parameters (increased specific volume, number of cells and height/width ratio, reduced density, average size, and perimeter of cells), antioxidant potential, and overall sensory quality of GFBs. This study showed an encouraging way of using a by-product that, due to its high content of proteins, polysaccharides, minerals, and antioxidants, can add value to GFBs.

## 1. Introduction

The food industry, including the plant oil industry, generates a great amount of food waste and by-products, which in many cases are not fully valorized, thus generating a major environmental problem [1]. The need to transform food processing by-products into useful ingredients is part of the circular economy and the zero-waste concept [2]. After processing, the agro-industrial waste can be used in the food, cosmetic, textile, and pharmaceutical industries. Moreover, some of these waste products and by-products can be considered a source of bioactive compounds, such as phenolic compounds, vitamins, pigments, oils, and various biopolymers such as polysaccharides (including dietary fiber) and proteins [3]. Press cakes, deriving from oilseeds extraction, represent interesting co-products due to their nutritional value, high biopolymers (proteins and polysaccharides) content, and the presence of bioactive phytochemicals, such as phenolic acids, flavonoids, lignans, and other antioxidant compounds [4,5,6]. The use of oilseed press cakes could be a sustainable alternative to reduce waste disposal and also contributes to the development of new, low-cost products rich in nutrients [1,7]. In fact, the application of various oilseed press cakes as a potential source for value addition in conventional food products is already reported [8,9]. Moreover, it should be emphasized that these by-products have improved nutritional value (elevated protein, fiber, macro- and microelements, polyphenols contents, and higher antioxidant capacities), as well as functional properties of gluten-free products [10].

Flaxseed (*Linum usitatissimum* L.) has emerged as an ample nutritional and functional food due to its appreciable amounts of high-quality proteins and minerals and exceptionally high content of α-linolenic acid, omega-3 fatty acid, lignans, and dietary fiber [11,12,13]. Numerous studies reported the production of high-quality flaxseed-enriched cereal products with the desired health attributes, exhibiting similar or improved shelf life compared to equivalent products [12]. For instance, Kaur et al. analyzed the effect of wheat flour replacement with flaxseed flour on the nutritional, functional, and antioxidant properties of cookies and reported that cookies produced with composite flour mixes were higher in protein, fat, ash, and fiber contents than control products [14]. Moreover, flaxseed-enriched cookies showed higher total polyphenolic content, antioxidant activity, as well as highly acceptable sensory scores. Similarly, Khouryieh and Aramouni evaluated the effect of flaxseed flour addition on the physical and sensory characteristics of cereal bars and indicated that flaxseed flour incorporation substantially enhanced the nutritional qualities of the cereal bars without affecting their sensory and quality properties [15]. After cold screw-pressed oil extraction, a flaxseed oil cake (FOC) is obtained [7]. This valuable and cheap by-product is underutilized in terms of food science and human food systems [16]. Only a few examples of FOC applications for the production of conventional foods are reported. For instance, Sanmartin et al. evaluated FOC as a tool for the improvement of the nutraceutical and sensorial features of sourdough bread [3]. Their results demonstrated that flaxseed cake-enriched sourdough bread could represent a potential vehicle for bioactive compounds with the possibility of obtaining high-quality products with improved nutritional profiles and desired health attributes. Similarly, Taglieri et al. reported that bread fortified with FOC showed a significant improvement in the nutraceutical profile and high antioxidant activity [7]. It can therefore be assumed that FOC has a high potential for use as a valuable additive in other food products.

Bakery products, including bread, are one of the most common staple foods willingly consumed all over the world every day [17]. However, some consumers cannot eat conventional wheat or wheat-rye products due to wheat/gluten-related disorders [18] or lifestyle, opting for gluten-free (GF) counterparts. Thus, the demand for high-quality GF products is continuously growing, and their market has become one of the most profitable segments of the food industry [19]. Many commercially available gluten-free breads (GFBs) have some disadvantages, such as unsatisfactory texture, low nutritional value, and short shelf life [20]. In addition, they are expensive and difficult to access. A characteristic feature of GFBs is their inability to develop a complex three-dimensional network due to a lack of gluten proteins (lack of gliadin and glutenin) [21]. Thus, to build up a network similar to that formed by gluten and mimic its viscoelastic properties (consequently, the appearance, quality, and sensory properties of bread-like products), the inclusion of other polymeric improvers is a critical factor [22]. Moreover, the additives should increase the water binding capacity at the dough/batter level resulting in increased loaf volume at the bread level [23]. Flours and/or starches of various origins (such as rice, corn, potato, and cassava), which are usually included in GFB formulations have a low structure-building ability and are often combined with binding agents such as proteins and hydrocolloids [24].

In this context, increasing attention is being paid to by-products and co-products deriving from flaxseed [3,12,13]. FOC is gluten-free; therefore, it could be used as an alternative raw material in GF-products, enriching them with a significant amount of proteins and minerals [25]. Flaxseed oil cake extract (FOCE) is a liquid matrix with unique properties due to its simultaneous flaxseed protein (FP) and flaxseed gum (FG) content, which is also abundant in antioxidants [26]. In previous works, it was demonstrated that strong FG and FP synergistic water holding and oil binding abilities make FOCE very a promising ingredient to stabilize food systems due to its high emulsifying [16,26,27] and encapsulating ability [28,29]. FG resembles functionally Arabic or guar gums more closely than other common gums, and it can be used to replace most non-gelling gums for food and non-food applications due to its ‘weak gel’-like property and remarkable water-holding capacity [26]. Flaxseed proteins are also investigated for their emulsifying properties [30]. FP and FG are also responsible for the formation of multiform structure and improved resistance to environmental stresses, causing FOCE to meet the requirements for both proteins and hydrocolloids in the context of GFB enhancement. Thus, based on the technological and nutritional features of FOCE, it could be potentially applied as a gluten replacer in GF breadmaking.

To the best of our knowledge, no studies have been carried out on GFB fortified with FOCE. We hypothesized that supplementation level could change the extent of influence exerted on GFB properties. Thus, the aim of the presented study was to produce, for the first time, FOCE-enriched GFBs and examine the influence of water replacement levels on their nutritional value, antioxidant properties, and sensory features.

## 2. Results and Discussion

The proximal chemical composition and energy value of experimental GFBs with FOCE replacing water in the gluten-free blend are presented in Table 1. The control GFB, composed mainly of starchy ingredients, was characterized by a low nutritional value, mainly due to the low proteins content (Table 1). A similar characteristic was noticed in other experimental GFBs composed of basic ingredients [31,32]. However, it should be emphasized that reduced nutritional adequacy is a worrisome trend observed in commercially available GFBs worldwide [20]. Comparing packaged gluten-free products to their regular gluten-containing counterparts, similar conclusions could be drawn [33,34]. These disadvantages negatively affect the nutrient status and health of patients on a strict gluten-free diet and therefore represent an urgent need to further efforts aimed to improve the nutritional quality of GFB.

In the presented research, a laboratory-produced FOCE was used [26], which is an extract from the post-production of flaxseed oil side-product, in order to improve the technical quality and nutritional value of GFB. Our research follows the sustainable food trend that is focused on the application of food processing side-products as a potential source of value addition to foods, resulting in novel foods of improved nutritional and nutraceutical value [9,31]. In fact, Taglieri et al. reported a significant improvement in the nutraceutical profile of the bread fortified with flaxseed cake in a dose-dependent manner [7]. Water substitution by FOCE in the experimental GFB formula caused a significant (*p* < 0.05) increase in protein content in the obtained products (Table 1), in particular, FOCE100% was 60% richer in proteins than the control. In addition, in breads enriched with the highest FOCE percentage (FOCE75% and FOCE100%), a significant (*p* < 0.05) increase in fat content was determined; however, in practice, this increase was relatively small and amounted to about 0.4 g/100 g (Table 1). On the other hand, the carbohydrate content in the samples containing FOCE was significantly reduced (*p* < 0.05).

Flaxseed proteins are known for their valuable amino acids composition. They are a source of arginine, aspartic acid, glutamic acid [35], cysteine, and methionine that were shown to improve the antioxidant status; thus, may have health-beneficial effects [36]. Changes determined in macronutrients content in experimental GFBs, in particular in proteins and fat, resulted directly from their relatively high content in FOCE (14 mg/mL of protein, 6.5 mg/mL carbohydrate, and 9.5 mg/mL of other extractable compounds) [27]. This had a direct impact on the energy value of the obtained baked goods (expressed in KJ and Kcal), which was lower compared to the control.

**Figure 1:**
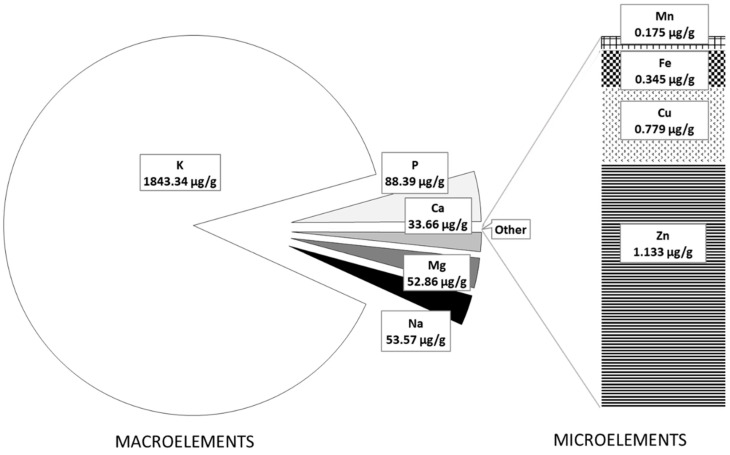
The macroelements and microelements content in FOCE.

**Figure 2:**
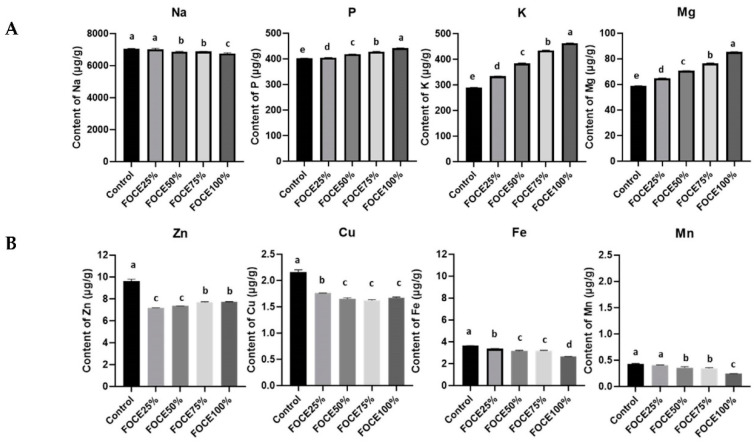
The minerals content in experimental gluten-free breads with FOCE: (**A**) macroelements (µg/g); (**B**) microelements (µg/g). Values with the same letter do not differ significantly (*p* < 0.05) when subjected to the one-way analysis of variance (ANOVA) with Tukey’s multiple comparison test.

The mineral composition of FOCE and the experimental GFBs is presented in Figure 1 and Figure 2, respectively. As expected, FOCE was a very rich source of potassium (K = 1843.34 µg/g) and contained a high amount of phosphorous (P = 88.387 µg/g) and magnesium (Mg = 52.86 µg/g) (Figure 1). Zinc (Zn) was the dominant microelement in FOCE, followed by copper (Cu) and small amounts of iron (Fe) and manganese (Mn) (Figure 1). The qualitative and quantitative minerals profile determined in FOCE reflects the characteristics of the raw material it derives from. According to the literature, flaxseed has a high amount of K (5600–9200 mg/kg) and is a good source of P (650 mg/100 g), Mg (350–431 mg/100 g), and Ca (Ca = 236–250 mg/100 g); however, it has a low amount of sodium (Na; 27 mg/100 g) [37,38].

The use of FOCE as a liquid component of the gluten-free blend resulted in the significant enrichment of GFBs in K. Its amount rose with the increasing FOCE to water ratio, and in FOCE100% K content was 60% higher than in the control (Figure 2A). High K intake is linked to improvements in cardiovascular diseases and is inversely related to blood platelet aggregation, free radicals in the blood, and stroke incidence [38]. Recently, Stone et al. [39] conducted a short-term clinical trial assessing the effect of increased K intake from different sources on blood pressure and microvascular outcomes in pre-hypertensive-to-hypertensive (systolic blood pressure > 120 mmHg) men and women. They observed a greater change in systolic blood pressure over time between the group on the K-rich diet (whose source were baked/cooked potatoes) compared with controls (−6.0 mmHg vs. −2.6 mmHg; *p* = 0.011) and concluded that increasing K intake might be beneficial for individuals with a higher risk of cardiometabolic diseases. Experimental GFBs with FOCE were characterized by a significantly (*p* < 0.05) higher content of Mg and P than the control bread (Figure 2A). On the other hand, the Na content decreased significantly with the increasing amount of FOCE in the experimental GFBs.

As shown by the analysis of atomic absorption spectrometry, FOCE was not abundant in the microelements (Figure 1). Among the analyzed microelements, only Zn content exceeded 1 µg/g, while the content of Cu, Fe, and Mn was very low. For this reason, the experimental GFBs with FOCE were characterized by a significantly (*p* < 0.05) lower amount of all micronutrients compared to the control bread (Figure 2B). Therefore, it is possible that other components of the bread formula and water were the main source of micronutrients in the GFB rather than FOCE itself.

**Figure 3:**
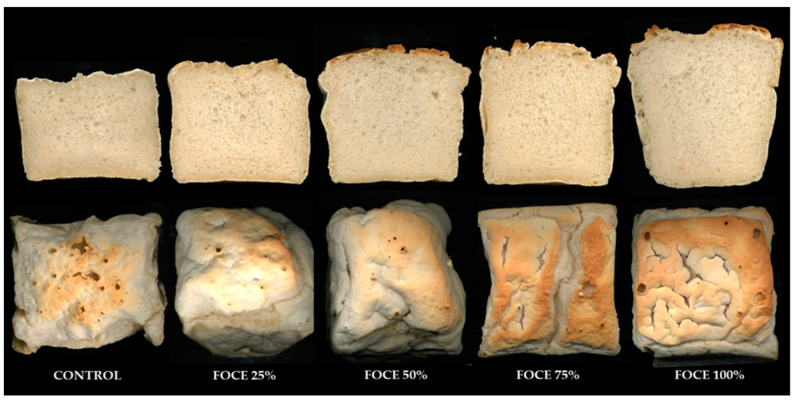
The exemplary pictures of the appearance of cross-section and surface of the experimental gluten-free breads with FOCE replacing water.

Technological parameters and the appearance of experimental GFBs are presented in Table 2 and Figure 3, respectively. The specific volume of the control bread was similar to results reported previously [31]; however, in comparison with a regular wheat bread, whose specific volume ranges from 3.5 to 5.5 cm^3^/g [40,41], the value of this parameter determined in the present study (2.39 cm^3^/g) was much lower. The use of FOCE in the experimental GFB formula resulted in a significant (*p* < 0.05) increase in the specific volume and height/width ratio, while the density of the obtained breads was reduced (Table 2). These changes were especially distinct in the case of the FOCE100%, whose specific volume was nearly 30% higher compared to the control, while its density was reduced by about 20%. The volume of bread representing the ability of the dough to expand without losing gas retention affects its specific volume, which is the primary determinant of the technological quality of the bread. The evidence obtained in the present study (Table 2) indicated that the increasing percentage of FOCE in the experimental formula promotes the quality of the obtained GFBs. The beneficial impact of FOCE on the technological features of GFBs could result from the chemical characteristics of FOCE. FOCE is derived from flaxseed, which besides being ample in nutrients, contains dietary fiber, in particular cellulose, mucilage gums, and lignin [37,38]. On the one side, the enrichment of regular bread with nutrient-dense and fiber-containing material due to possible gluten dilution, competitive water-binding [42] or physical disruption of the gas cells and gluten network [43] was shown to have a detrimental effect on the dough viscoelastic properties and bread volume. On the other side, soluble fibers of flaxseed had positive effects on the wheat dough structure and loaf volume [44,45]. The high value of specific volume, together with the proper aeration of GFBs crumb resulting from relatively small pores regularly distributed across the crumb (Figure 3), are required to obtain products of satisfactory sensorial quality [3]. In fact, FOCE increased the number of cells in GFBs. In addition, compared to the control sample, there was a significant reduction in the average cells area and perimeter but an increase in their circularity in FOCE-enriched GFBs. This observation is in line with the results reported by Aranibar et al. [9], who analyzed the influence of chia by-products on the structure of wheat muffins. Sabanis and Tzia [46] indicated that soluble fibers of a high water-binding capacity improve the retention of water during dough mixing, which evaporates during baking, increases the internal pressure, and consequently increases the volume of the loaf. Therefore, an improvement in the technological parameters of experimental GFBs could result from the functional ingredients of FOCE. Drozłowska et al. reported that FOCE has high stabilizing potential due to the different water-holding and oil-binding capacities of flaxseed gum and protein and the effective decreasing of interfacial tension [26]. This probably facilitated the creation and entrapping of carbon dioxide in the pores during baking. Moreover, as the GFBs formulations also contained oil, their enhanced technological features can also be presumably linked with the emulsifying activity of FP, as proteins preferentially adsorb to the oil–water interfaces and form a viscoelastic film, which provides physical stability to the emulsions during their subsequent processing and storage [26,27]. Furthermore, the baking process takes place at elevated temperatures, during which the thermal partial or complete denaturation of proteins can occur, depending on the temperature level and exposure time. The main process causing denaturation is the exposure of previously unexposed hydrophilic molecules and sulfhydryl groups to water. These groups are responsible for hydrophobic interactions with oil phases, while hydrophilic amino acid residues located on the surface can absorb water. Due to this structure, which is the result of controlled heat treatment, FP can interact with both oil and water phases and can act as an effective emulsifying agent to stabilize the phase interfaces, similar to some other heat-modified proteins [27]. In fact, it was shown that denatured FOCE has a higher ability to stabilize oil-containing systems than the native one [47].

The results of the instrumental color analysis conducted in the control and GFBs enriched with FOCE are presented in Table 2. Due to the very uneven and inhomogeneous crust surface of the experimental breads (Figure 3), only the crumb color was analyzed. The crumb of the control bread was light (L* = 71.29) and of creamy-to-beige color. This result corresponds well with the results of our earlier study (L* = 71.58) [31]. The starch-based GFBs usually have a whitish crumb and light-colored crust perceived as pale and unattractive when compared with regular wheat bread [48]. According to the instrumental color analysis, FOCE had a significant impact on the color of the crumb of experimental GFBs. As reported in Table 2, FOCE100% showed the highest values of both lightness (L*), as well as the blue–yellow components (b*) and red–green components (a*). All these parameters increased as a function of the concentration of FOCE in the bread formula. This observation is in agreement with the results of Tavarini et al. [36], who also reported that the color of the crumb of bread was significantly affected by FOC addition. The shade of the crumb depends mainly on the ingredients used in the formulation. FOCE was of a slightly creamy-to-beige liquid (data not shown); therefore, apparent differences in crumb color were not easily distinguished by the human eye (Figure 3). However, when the metric distances among the coordinates were calculated (ΔE) [49], the hue of the crumb of the control bread was evidently different (1 < ΔE < 3 or ΔE > 3) from the hue of the FOCE-enriched GFBs crumbs (Table 2).

The content of the total phenolic content (TPC) and the antioxidant power were analyzed in both FOCE and GFBs with different percentages of FOCE, and the obtained results are reported in Table 3. It was not surprising that FOCE, due to the production method, was characterized by a relatively low content of TPC (0.16 mg GAE/g DM) when compared to flaxseed cake [7] and whole flaxseed flour [14]. Nevertheless, FOCE was characterized by the antioxidant capacity that was confirmed by ABTS, DPPH, and FRAP assays, while not by PCL-ACW (Table 3). On the other hand, FOCE showed the PCL-ACL activity which is associated with the lipophilic antioxidants (Table 3), possibly fat-soluble vitamins (A and E). γ-tocopherol is an antioxidant providing protection to cell proteins and fat from oxidation and is related to the reduced risk of Alzheimer’s disease [50].

The TPC content of GFBs containing FOCE doubled compared to the control (Table 3), but the increase noted was not directly related to the amount of FOCE replacing water in the gluten-free blend. While the increase of the antioxidative activity, determined by ABTS, DPPH, FRAP, and PCL-ACW assays, was proportional to the level of FOCE in the GFB formulas. In particular, FOCE100% showed the highest antioxidant power in comparison to the control (Table 3). Results obtained in the present study are in accordance with observations by Sanmartin et al. [3], who investigated the nutraceutical properties of wheat bread fortified with FOC. They found that the antioxidant power significantly increased (*p* < 0.001) with the growing percentage of flaxseed cake added to the flour mix. Additionally, Man et al. [51], in their study, aimed to examine the effects of partial replacement of wheat flour with roasted flaxseed flour and found that the total phenolic content and the antioxidant activity increased with increasing amounts of the roasted flaxseed flour in the biscuits. Taglieri et al. [7] also reported an increased level of total phenols and anti-radical activity in FOC-enriched bread.

The FOCE used in the present study was characterized by moderate antioxidant activity, but it turned out to be a valuable material influencing the bioactive characteristics of GFBs. The enhanced antioxidant capacity detected in FOCE-enriched GFBs could potentially result from FOCE composition [27,29]. On the other side, the whole flaxseed is known to be an excellent source of lignans (predominantly secoisolariciresinol diglucoside) and other phenolic compounds (ferulic acid, syringic acid, cinnamic acid, vanillic acid, *p*-coumaric acid, and gallic acid) of antioxidative properties [52,53,54]. Secoisolariciresinol diglucoside lignan-enriched flaxseed powder was demonstrated to reduce body weight and fat accumulation, improve the lipid profile, and lower blood pressure in the animal model [55]. Moreover, the population-based case–control observational study [56] reported that the consumption of rich in lignans flaxseed was associated with reduced breast cancer risk. On the other hand, substracts delivered by FOCE to the GFB matrix became the reagents of the Maillard reaction. In general, this non-enzymatic browning reaction involves two main types of reactants, reducing sugars and amino acids; however, the condensation reactions between amino acids and lipid oxidation products may also form Maillard reaction products (MRPs), and the role of lipids in the Maillard reaction is similar to the role of reducing sugars [57]. MRPs, especially melanoidins, are reported to have antioxidant activity [58]. Therefore, FOCE used as liquid replacing water in the GFB formula can promote the formation of MRPs during baking. In fact, the elevated temperature is reported to promote MRPs formation in FOCE during spray-drying [27,29]. The bread crust present in the analyzed samples, while making up only a small fraction of the total bread weight, was the major contributor to the observed increase in the antioxidant capacity of GFBs with FOCE.

**Figure 4:**
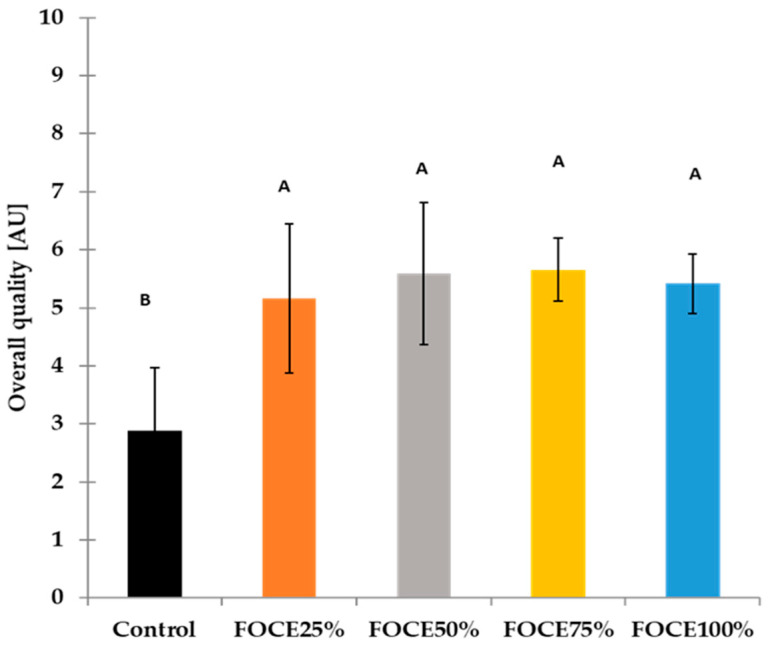
The overall sensory quality of gluten-free breads with FOCE. Different letters (A, B) above the bars mean that values differ significantly (*p* < 0.05), as determined by the one-way analysis of variance (ANOVA) with Tukey’s multiple comparisons test.

Experimental GFBs were subjected to quantitative descriptive analysis (QDA), the results of which are presented in Table 4 and Figure 4. The FOCE used in the bread blend significantly influenced (*p* < 0.05) the appearance of the analyzed breads. Regardless of their amount, all GFBs containing FOCE were characterized by a regularly distributed pore collocation (from 7.17 to 7.73 AU) in comparison to the control (2.57) (Table 4). Moreover, the color of the crumb of GFBs with FOCE was more beige. For the pore dimension, no significant difference was encountered between the control sample and GFBs with lower FOCE percentages (FOCE25% and FOCE50%); however, in samples with a higher FOCE to water ratio (FOCE75% and FOCE100%) the dimension of the pores was 40–60% bigger than in the control. Although crumb cellular structure has a significant effect on the bread quality, its mechanical properties are weakly dependent on cell size while being influenced by the distribution of cells [59]. As indicated in Table 4, the control bread was of lower quality (lower specific volume and denser crumb) than the GFBs with FOCE. The beneficial effect of FOCE used in the experimental formula was proven by an enhancement in the technological properties (Table 2) and was additionally reflected in the visual structure of the crumb (Figure 3) with evenly collocated pores (Table 4).

When assessing the odor features of the experimental GFBs, panelists identified four odor attributes: acid, oily, wheat bread, and sweet. All GFBs were perceived as similarly slightly sweet (ranging from 1.33–1.49 AU) and oily (approximately 2 AU), and their odor resembled wheat bread (Table 4). The only difference between the samples resulted from an acid odor that was significantly less intensive (*p* < 0.05) in the GFBs containing higher levels of FOCE (FOCE75% and FOCE100%) in comparison to the control. The fresh flaxseed had a unique, slightly nutty flavor [60], which could be pleasant for the consumers; however, this odor was not detected in the FOCE-enriched GFBs. Nevertheless, the odor of the obtained baked goods improved when FOCE was used in the formula. Assessing the taste, panelists indicated that GFBs containing FOCE were perceived as significantly less salty than the control (Table 4). This results directly from the low sodium content of FOCE and its decreasing content in GFBs with increasing FOCE content (Figure 2). In contrast, the acid taste, which was barely perceptible in the control (1.54 AU), was more distinct in GFBs with FOCE. In particular, the higher the ratio of FOCE to water in the gluten-free mixture, the more intense the acid taste was perceived. This observation is in line with the results of Taglieri et al. [7], who reported increased acidity in FOC-fortified breads due to the presence of unsaturated free fatty acids.

The texture plays a key role in the consumers’ preferences for foods. In the present study, GFBs were evaluated through their behavior in the mouth while eaten and the elasticity while pressing by finger. The texture parameters evaluated in the mouth (chewiness, adhesiveness, moisture) did not differ meaningfully for all experimental breads, while when bread crumb was pressed by a finger, the GFBs with FOCE were more elastic than the control (Table 4). This can be related to the relatively high proteins content in FOCE (Table 1) and probably to the soluble fibers that may bind water, thus influencing the textural attributes of the obtained product. Although compared to the control, some sensory attributes of the GFBs with FOCE deteriorated (acid taste), the appearance, aroma, and texture resulted in a high overall appreciation of GFBs with FOCE (Figure 4). Among the experimental GFBs with FOCE, the highest scores in overall quality obtained FOCE75% (5.66 AU). This result was almost twice as high in comparison to the control bread (2.88 AU). Taking into account the above results of the sensory analysis, it can be concluded that the higher level of FOCE in the GFB formula allows us to obtain a product with a favorable appearance and higher sensory quality compared to both the control containing only water and the bread with the lower content of FOCE (FOCE 25% and FOCE 50%).

## 3. Materials and Methods

### 3.1. Preparation of Flaxseed Oil Cake Extract (FOCE)

FOCE was prepared according to the previously developed procedure described in the previous study [26]. Briefly, FOC (ACS Sp. z o.o., Bydgoszcz, Poland) was extracted with hot distilled water (1:10 *w*/*w*, 90 °C, 1 h, 250 rpm), then cooled down to 20 °C and centrifuged (4000 rpm, 30 min) to obtain FOCE. Subsequently, FOCE was filtered and homogenized (12,000 rpm, SilentCrusherM, Heidolph, Germany).

### 3.2. Preparation of Experimental Gluten-Free Bread

A previously developed formulation of gluten-free bread (GFB) [31] was used as a control. Corn starch (HORTIMEX, Konin, Poland), potato starch (PPZ “Trzemeszno” Sp. Z o.o., Trzemeszno, Poland), sugar, fresh yeast (Lesaffre Polska S.A., Wołczyn, Poland), pectin (E 440(i), ZPOW Pektowin, Jasło, Poland), rapeseed oil “Kujawski” (ZT “Kruszwica” S.A., Kruszwica, Poland), salt, and water were the main ingredients of GFB (Table 5.) FOCE was incorporated into the experimental GFBs as a liquid that replaced from 25 to 100% (*v*/*v*) of the water in the control GFB formulation.

All solid ingredients (corn starch, potato starch, pectin) were mixed (5 min) at minimum speed using a laboratory planetary mixer (KitchenAid Professional K45SS mixer; KitchenAid Europa, Inc., Brussels, Belgium) in the stainless steel bowl with a flat beater. The remaining ingredients (salt, sugar, and yeast) were dissolved separately in the water and added to the dry mixture, together with oil. Then, the batter was mixed for 12 min at speed 2. A 240 g sample of the resulting batters was placed in a hexagon-shaped bread pan (10 cm × 10 cm × 9 cm, length, width, and height, respectively), covered with baking paper, and proofed for 40 min in a proofing cabinet (35 °C, 70% humidity). Experimental GFBs were baked (220 °C, 30 min) in the laboratory oven (ZBPP, Bydgoszcz, Poland). The obtained bread loaves were cooled for 2 h at room temperature, and then they were packed in clip-on plastic bags and kept in the dark at room temperature for further analysis. The products of two independent batches were analyzed.

### 3.3. Characteristics of Experimental GFBs

#### 3.3.1. Determination of the Bread Quality

The experimental GFBs were evaluated 24 h after baking as then a better differentiation could be realized. Three loaves of each kind of GFBs were analyzed. The weight of the GFBs was evaluated using a digital balance with 0.01 g accuracy. The loaf volume was determined using a modified standard rapeseed displacement method, in which millet seeds were used instead of rapeseed.

Specific volume (SV) was calculated as the loaf volume divided by its weight. Density (D) was calculated as the loaf weight divided by its volume. Bake loss was calculated as indicated in Equation (1):

(1) $$\mathrm{Bake}\;\mathrm{loss}\;\left(\%\right)=\frac{\left(a-b\right)\times 100}{a}$$

where:

*a*—the weight of batter (g),

*b*—the weight of baked and cooled GFBs (g).

The height/width ratio of the GFBs was determined at their middle slice. A scan of the example central slice of each experimental GFs was obtained using a flatbed scanner (Epson Perfection V200 Photo) supported by Epson Creativity Suite Software Images.

#### 3.3.2. Evaluation of Bread Crumb Porosity

The crumb porosity and distribution of air cells were examined employing image analysis following the procedure described by Aranibar et al. [9]. A photo scanner (Epson GT-1500, Epson Europe B.V. Sp. z o.o. Warsaw, Poland) was used to take the images where the GFBs were cut transversely. Images in JPEG format were analyzed using Image-J image analysis software. A representative area of equal size was manually selected for all crumbs. The color image was converted to an 8-bit image and analyzed in grayscale. Segmentation of the image (conversion to a binary image) was performed by the program by automatically selecting a threshold value. The following parameters were obtained from the image analysis: the number of cells (No/cm^2^), average cells area (mm^2^), average cell perimeter (mm), and cell circularity (1 = maximum circularity, 0 = without circularity).

#### 3.3.3. Instrumental Bread Crumb Color Determination

The color of the crumb of GFBs was analyzed at the middle point of the central 2 cm slice using a HunterLab ColorFlex (Hunter Associates Laboratory, Inc., Reston, VA, USA). The measurements were performed through a 3 cm diameter diaphragm containing an optical glass. The color was expressed in accordance with the CIELab system and the parameters determined were: lightness (L* = 0 [black] and L* = 100 [white] and chromatic components: a* (–a* = greenness and +a* = redness) and b* (–b* = blueness and +b* = yellowness). The Δ*E_Lab_* difference between two colors [49] was calculated according to Equation (2):

(2) $$\Delta E_{Lab}=\sqrt{{\left(\Delta L^{*}\right)}^{2}+{\left(\Delta a^{*}\right)}^{2}+{\left(\Delta b^{*}\right)}^{2}}$$

where: ΔL* = *L*1 − *L*0; Δa* = *a*1 − *a*0; Δb* = *b*1 − *b*0.

Values of the crumb color were the mean of at least six replications.

#### 3.3.4. Determination of the Proximal Chemical Composition and Energy Value

Moisture (method 926.05) was determined in the fresh GFBs, while the content of proteins (method 979.09), fat (method 923.03), and total ash (method 930.22) were determined in freeze-dried experimental GFBs according to the standard method [61]. The total carbohydrate content was calculated by subtracting the values of the moisture, protein, fat, and ash content from 100. The energy values (kJ) were calculated by multiplying the amount of macronutrient by the corresponding conversion factors (17 kJ/g for protein, 37 kJ/g for fat and 17 kJ/g for carbohydrates) [28]. The conversion factors for calories calculation is: 1 kJ = 0.239 kcal [62].

#### 3.3.5. Determination of Mineral Concentration

The concentration of the selected minerals in the FOCE and GFBs was analyzed by a flame (air—acetylene burner) atomic absorption spectrometry method (AAS) using an atomic absorption spectrophotometer (iCE 3000 Series, Thermo Fisher Scientific Inc., Loughborough, United Kingdom). Before analysis, the samples were wet-digested with a mixture (9:1; *v*/*v*) of concentrated nitric acid (65% HNO_3_; Merck, Darmstadt, Germany) and hydrochloric acid (30% HCl; Merck) using a microwave system (Multiwave, Anton Paar GmbH, Graz, Austria). For analytical quality control, analyses were repeated three times.

### 3.4. Antioxidant Capacity of FOCE and GFBs

#### 3.4.1. Sample Preparation and Antioxidants Extraction

The whole experimental GFBs and FOCE were subjected to a freeze-drying process. To obtain extracts, about 200 mg of GFBs or 100 mg FOCE samples were weighed into microcentrifuge tubes and extracted with 1 mL of 80% aqueous methanol. Each stage of five-fold extraction consisted of 30 s sonication (VC 750, Sonics & Materials, Inc., Newtown CT, USA) and subsequent 30 s vortexing and centrifugation at 13,200× *g* for 10 min at 4 °C (5415R centrifuge, Eppendorf, Germany). The obtained supernatants were collected into a 5 mL measuring flask. After that stage, 1 mL of solvent was added to the remaining pellets, and the extraction was continued up to five times.

#### 3.4.2. Total Phenolic Content (TPC)

The TPC was determined according to the method described previously [31] with Folin–Ciocalteu reagent that was freshly diluted with water in proportion 1:15 (*v*/*v*). The TPC assay was performed in microplates, and aliquots of 15 μL of methanol extracts were placed in microplate wells. Subsequently, 250 μL of the Folin–Ciocalteu solution were added, and the mixture was incubated in the dark for 10 min at room temperature. Then, 25 μL of 20% sodium carbonate were added to each well, and the mixture was incubated for 20 min. The microplate was shaken automatically before readings, and absorbance was measured at λ = 755 nm (Infinite M1000 PRO plate reader; Tecan Group AG, Männedorf, Switzerland). Gallic acid was used for standard calibration (0.03–1.0 mg/L), and the results were expressed in mg of gallic acid equivalents (GAE) per one gram of dry matter (g/DM) of GFBs or FOCE.

#### 3.4.3. ABTS Assay

The Trolox Equivalent Antioxidant Capacity (TEAC) was performed as described by Horszwald and Andlauer [63]. 2,2′-azino-bis(3-ethylbenzothiazoline)-6-sulphonic acid) radical cation (ABTS^·+^) was prepared by reacting 7 mM ABTS aqueous solution with 51.4 mM potassium persulfate and allowing the mixture to stand in the dark at room temperature for 12–17 h before use. The working solution of the radical cation was prepared directly before analysis by diluting the stock solution with 80% (*v*/*v*) methanol to obtain an absorbance value of 0.7 ± 0.02 at λ = 734 nm. For analysis, 10 μL of the samples (the methanol extracts of GFBs and FOCE prepared as described in paragraph 2.3.1), blank (80% *v*/*v* methanol) or standard were put into the microplate wells. Subsequently, 290 μL of ABTS·+ working solution were added to each well, and the plate was put into the microplate reader Infinite M1000 PRO (Tecan Group AG, Männedorf, Switzerland), shaken gently for 5 s, and left at 30 °C in the dark for 6 min followed by absorbance measurement at λ = 734 nm. ABTS^+^ scavenging effect (% inhibition) was calculated according to Equation (3):

(3) $$\%\;\mathrm{inhibition}=\frac{\left(A0-A1\right)\;}{A0}\times 100$$

where:

A0—the absorbance value of the blank sample,

A1—the absorbance of tested samples.

A synthetic analogue of vitamin E, 6-Hydroxy-2,5,7,8-tetramethylchroman-2-carboxylic acid (Trolox), was used as an antioxidant standard in the concentration range between 10–750 μM. The results were expressed in μmol Trolox/g DM of GFBs or FOCE.

#### 3.4.4. DPPH Assay

The 2,2-diphenyl-2-picrylhydrazyl (DPPH) radical scavenging assay was performed according to Brand-Williams et al. [64]. The 2,2-diphenyl-2-picrylhydrazyl radical (DPPH·) solution was prepared freshly before analysis by dissolving 10 mg DPPH in 250 mL of 80% (*v*/*v*) methanol. The obtained radical solution provided an absorbance value in the range from 0.95 to 1.10 at λ = 517 nm. For analysis, 20 μL of methanol extracts of experimental GFBs and FOCE (as described in paragraph 2.3.1), blanks or standard were placed into 96-well microtitre plates, and then DPPH· solution (300 μL) was added. The reaction mixtures in the plate were incubated at ambient temperature for 30 min in the dark. Then, absorbance was recorded at λ = 517 nm by the microplate reader Infinite M1000 PRO (TecanGroup AG, Männedorf, Switzerland). Trolox was used for standard calibration (0.005–0.75 mM). The values were the means of triplicate analyses and expressed as μmol Trolox/g DM of GFBs or FOCE.

#### 3.4.5. FRAP Assay

A ferric reducing antioxidant potential (FRAP) assay was carried out according to the microplate method described by Benzie and Strain [65]. Briefly, the FRAP reagent was prepared directly before analysis by mixing 5 mL of 10 mM 2,4,6-tri(2-pirydyl)-s-triazine (TPTZ) in 40 mM hydrochloric acid with 5 mL of 20 mM ferric (III) chloride solution and with 50 mL of 300 mM acetate buffer (pH = 3.6). Fifty µL of appropriately diluted extracts were added directly to the 96-well microplate, followed by a 300 µL FRAP reagent. The microplate was put into the microplate reader Infinite M1000 PRO (TecanGroup AG, Männedorf, Switzerland), shaken gently for 5 s, and left at ambient temperature in the dark for 5 min, followed by absorbance measurement at λ = 593 nm. Trolox was used as an antioxidant standard in the concentration range between 10 and 100 μM, and the results were expressed as µmol Trolox per 1 g of dry matter of the samples. All the determinations were performed in triplicate.

#### 3.4.6. Photochemiluminescence Assay

A photochemiluminescence (PCL) assay was performed as described by Zieliński, Zielińska, and Kostyra [66]. This method was used to estimate the antioxidant activity of the experimental samples against superoxide anion radicals generated from luminol (photosensitizer) under exposure to UV light in the Photochem apparatus (Analytik Jena, Leipzig, Germany). PCL-ACW (hydrophilic condition) and PCL-ACL (lipophilic condition) determinations were carried out using the analytical kits offered by the manufacturer (ACW 400.801 and ACL 400.802) and in accordance with the application protocols. For PCL-ACW, a 50 mg sample was extracted with 1 mL of water, and for PCL-ACL, a 50 mg sample was extracted with 1 mL of the methanol and hexane mixture (4:1; *v*/*v*). PCL-ACW studies were performed by a 180 s measurement and automatic calculation of the lag time when chemiluminescence was not generated due to the antioxidants present in extracts. The difference between the lag time of the tested sample and the mean of the blanks was the parameter of radical-scavenging activity conversed to antioxidant capacity by comparison with the Trolox standard curve within the range of 0.5–3 nmol. In PCL-ACL analysis, chemiluminescence emitted by luminol after excitation by free radicals that remain in the measuring cell after the reaction with the antioxidant present in tested samples was registered for 180 s. The integral under the signal curves of the tested sample and the mean of the blanks were calculated automatically, followed by a comparison with the Trolox standard curve (0.25–3 nmol). The results of PCL-ACW and -ACL were expressed in μmol Trolox/g DM. The total antioxidant capacity was also calculated by adding the values of the PCL-ACW and PCL-ACL antioxidant activities.

### 3.5. Sensory Evaluation

Sensory analysis was carried out by an expert panel consisting of six non-celiac assessors (five women and one man) who were familiar with gluten-free products. They were trained according to ISO guidelines [67]. The sensory characteristics of experimental GFBs were evaluated 24 h after baking. A quantitative descriptive analysis (QDA) [68] was applied to assess the sensory quality of the GFBs. Before the analysis, panelists determined the vocabularies of the sensory attributes in a round-table session using a standardized procedure [69]. Seventeen attributes were evaluated (Table 6). The assessors evaluated the intensity perceived for each sensory attribute on unstructured graphical scales. The scales were 10 cm long and verbally anchored at each end, and the results were converted to numerical values (from 0 to 10 arbitrary units) by a computer. The experimental GFB samples were coded with a three-digit number and presented to the assessors all together in a random order in transparent plastic boxes. The sensory evaluation was carried out in a sensory laboratory room, which fulfills the requirements of the ISO standards [70], under normal lighting conditions at room temperature. To minimize residual effects, it was suggested to drink bottled mineral water between each sample evaluation. The results were collected using a computerized system ANALSENS (IAR&FR PAS, Olsztyn, Poland). GFBs were tested in two replications.

### 3.6. Statistical Analysis

Unless otherwise stated, the data reported in tables are presented as mean values and standard deviations of triplicate observations. The differences between experimental GFBs were analyzed by a one-way analysis of variance (ANOVA) with Tukey’s multiple comparison test (*p* < 0.05) using GraphPad Prism version 8.0.0 for Windows, GraphPad Software (San Diego, CA, USA).

## 4. Conclusions

On the basis of the obtained results, it can be concluded that FOCE can successfully replace the water in the GFB formula as a substitute with a positive effect on the quality of the developed baked product. The use of FOCE resulted in an improvement in the nutritional value of the bread, which was thus enriched with protein and minerals, especially K and Mg. Replacing water with FOCE also resulted in the improvement of the technological parameters of the bread, especially in the samples with a high proportion of FOCE to water. All these beneficial changes caused by the use of FOCE had an impact on the improvement of sensory quality. It should also be emphasized that FOCE significantly improved the antioxidant potential of GFB. In summary, the obtained GFB with FOCE, especially FOCE75%, can be seen as an added value product that may enrich the daily diet of consumers with valuable nutrients as well as ingredients with pro-health potential.

## Figures and Tables

**Table 1:** The nutritional and energy value of the gluten-free breads with FOCE replacing water.

	Control	FOCE25%	FOCE50%	FOCE75%	FOCE100%
Moisture (%)	57.71 ^a^ ± 1.08	62.04 ^a^ ± 1.15	61.13 ^a^ ± 1.32	60.91 ^a^ ± 0.94	60.46 ^a^ ± 1.82
Proteins (g/100 g DM)	1.30 ^d^ ± 0.01	1.52 ^c^ ± 0.05	1.64 ^c^ ± 0.07	1.84 ^b^ ± 0.01	2.08 ^a^ ± 0.01
Ash (g/100 g DM)	1.72 ^a^ ± 0.08	1.72 ^a^ ± 0.04	1.71 ^a^ ± 0.01	1.68 ^a^ ± 0.04	1.77 ^a^ ± 0.03
Fat (g/100 g DM)	2.37 ^c^ ± 0.06	2.49 ^c^ ± 0.02	2.60 ^bc^ ± 0.05	2.79 ^a^ ± 0.06	2.74 ^ab^ ± 0.03
Carbohydrates * (g/100 g DM)	36.90 ^a^ ± 0.05	32.23 ^c^ ± 0.06	32.96 ^b^ ± 0.03	32.78 ^b^ ± 0.05	32.90 ^b^ ± 0.12
Energy value (kJ)	737	666	684	692	696
Energy value (kcal)	174	157	162	164	165

* Calculated from the difference. DM—Dry Matter. Within each row and for each factor, values with the same letter (^a, b, c, d^) do not differ significantly (*p* < 0.05) when subjected to the one-way analysis of variance (ANOVA) with Tukey’s multiple comparison test.

**Table 2:** The technological quality parameters of gluten-free breads with FOCE replacing water.

	Control	FOCE25%	FOCE50%	FOCE75%	FOCE100%
Specific volume (cm^3^/g)	2.39 ^c^ ± 0.03	2.39 ^c^ ± 0.04	2.78 ^b^ ± 0.03	2.87 ^b^ ± 0.01	3.06 ^a^ ±0.09
Bake loss (%)	12.23 ^a^ ± 0.41	12.00 ^a^ ± 0.15	12.17 ^a^ ± 0.21	12.22 ^a^ ± 0.10	11.75 ^a^ ± 0.41
Density (g/mL)	0.42 ^a^ ± 0.01	0.42 ^a^ ± 0.01	0.35 ^b^ ± 0.01	0.36 ^b^ ± 0.01	0.33 ^c^ ± 0.01
Height/width ratio	1.06 ^b^ ± 0.03	1.06 ^b^ ± 0.05	1.10 ^b^ ± 0.06	1.12 ^b^ ± 0.06	1.22 ^a^ ± 0.05
Crumb cells parameters					
Number of cells (No/cm^2^)	28 ^b^ ± 4	41 ^a^ ± 5	41 ^a^ ± 3	44 ^a^ ± 1	44 ^a^ ± 5
Average cells area (mm^2^)	6.66 ^a^ ± 1.61	3.55 ^b^ ± 1.03	3.91 ^b^ ± 0.37	4.00 ^b^ ± 0.94	3.93 ^b^ ± 1.33
Average cell perimeter (mm)	27.61 ^a^ ± 0.54	15.24 ^b^ ± 2.34	17.04 ^b^ ± 2.20	15.50 ^b^ ± 1.14	13.07 ^b^ ± 3.34
Cell circularity (-)	0.09 ^b^ ± 0.01	0.20 ^a^ ± 0.03	0.24 ^a^ ± 0.05	0.20 ^a^ ± 0.03	0.26 ^a^ ± 0.07
Crumb color					
L*	71.29 ^c^ ± 1.76	71.10 ^c^ ± 0.76	74.13 ^b^ ± 0.68	73.67 ^b^ ±0.94	76.02 ^a^ ± 0.91
a*	1.46 ^a^ ± 0.14	1.08 ^d^ ± 0.15	1.13 ^cd^ ± 0.04	1.26 ^bc^ ± 0.09	1.31 ^b^ ± 0.10
b*	16.95 ^b^ ± 0.81	13.86 ^c^ ± 0.94	17.17 ^b^± 0.44	16.83 ^b^ ± 1.17	18.35 ^a^ ±0.59
Δ*E*	Served as control	3.12	2.86	2.39	4.93

Within each row and for each factor, values with the same letter (^a, b, c, d^) do not differ significantly (*p* < 0.05) when subjected to the one-way analysis of variance (ANOVA) with Tukey’s multiple comparison test.

**Table 3:** The total phenolic content (mg GAE/g DM) and antioxidant capacity (µmol TE/g DM) of FOCE and gluten-free breads with FOCE.

	FOCE	Control	FOCE25%	FOCE50%	FOCE75%	FOCE100%
TPC (mg GAE/g DM)	0.162 ± 0.011	0.096 ^b^ ± 0.004	0.203 ^a^ ± 0.029	0.222 ^a^ ± 0.021	0.232 ^a^ ± 0.009	0.234 ^a^ ± 0.008
ABTS (µmol TE/g DM)	1.321 ± 0.035	0.792 ^d^ ± 0.050	0.890 ^c^ ± 0.040	0.985 ^bc^ ± 0.038	1.076 ^ab^ ± 0.029	1.128 ^a^ ± 0.010
DPPH (µmol TE/g DM)	0.987 ± 0.053	0.886 ^ab^ ± 0.026	0.852 ^b^ ± 0.030	0.863 ^ab^ ± 0.040	0.882 ^ab^ ± 0.044	0.945 ^a^ ± 0.020
FRAP (µmol TE/g DM)	0.794 ± 0.013	0.435 ^c^ ± 0.016	0.575 ^b^ ± 0.020	0.568 ^b^ ± 0.011	0.731 ^a^ ± 0.016	0.703 ^a^ ± 0.014
ACW (µmol TE/g DM)	N/A	0.158 ^b^ ± 0.009	0.147 ^b^ ± 0.009	0.166 ^ab^ ± 0.009	0.184 ^a^ ± 0.006	0.184 ^a^± 0.008
ACL (µmol TE/g DM)	0.675 ± 0.011	0.100 ^a^ ± 0.004	0.089 ^b^ ± 0.002	0.091 ^b^ ± 0.002	0.094 ^ab^ ± 0.003	0.098 ^a^ ± 0.001
PCL * (µmol TE/g DM)	0.675	0.258	0.236	0.257	0.278	0.282

Within each row, and for each factor, values with the same letter (^a, b, c, d^) do not differ significantly (*p* < 0.05) when subjected to the one-way analysis of variance (ANOVA) with Tukey’s multiple comparison test; * PCL—calculated as a sum of ACW and ACL. GAE—Gallic Acids Equivalents, TE—Trolox Equivalents, DM—Dry Matter.

**Table 4:** The sensory characteristics of gluten-free breads with FOCE.

		Control	FOCE25%	FOCE50%	FOCE75%	FOCE100%	*p*-Value
Appearance	beige color	1.31 ^b^	1.71 ^a^	1.73 ^a^	1.85 ^a^	1.71 ^a^	0.0005
	pore collocation	2.57 ^b^	7.17 ^a^	7.53 ^a^	7.57 ^a^	7.73 ^a^	<0.0001
	pore dimension	1.41 ^b^	1.63 ^ab^	1.81 ^ab^	2.24 ^a^	1.98 ^a^	0.0084
Odor	acid	2.35 ^a^	1.46 ^ab^	1.48 ^ab^	1.21 ^b^	1.23 ^b^	0.0038
	oily	2.15 ^a^	2.17 ^a^	2.27 ^a^	2.21 ^a^	2.03 ^a^	0.9445
	wheat bread	1.61 ^a^	2.00 ^a^	1.79 ^a^	2.07 ^a^	2.10 ^a^	0.2213
	sweet	1.45 ^a^	1.42 ^a^	1.33 ^a^	1.44 ^a^	1.49 ^a^	0.8731
Texture (manual)	elasticity	5.3 ^b^	6.59 ^a^	6.54 ^a^	6.51 ^a^	6.59 ^a^	0.0018
Texture (in the mouth)	chewiness	2.23 ^a^	1.93 ^a^	1.89 ^a^	1.82 ^a^	1.98 ^a^	0.3161
	adhesiveness	1.67 ^a^	1.70 ^a^	1.45 ^a^	1.45 ^a^	1.47 ^a^	0.7294
	moisture	2.93 ^a^	2.83 ^a^	2.89 ^a^	2.93 ^a^	2.83 ^a^	0.9861
Taste	wheat bread	1.82 ^a^	2.16 ^a^	2.23 ^a^	2.45 ^a^	2.18 ^a^	0.1201
	acid	1.54 ^c^	2.01 ^bc^	2.36 ^abc^	2.69 ^ab^	3.04 ^a^	0.0002
	sweet	1.27 ^a^	1.87 ^a^	1.80 ^a^	1.77 ^a^	1.82 ^a^	0.0671
	salty	0.68 ^a^	0.45 ^b^	0.46 ^b^	0.48 ^b^	0.42 ^b^	0.0059
	aftertaste	2.92 ^a^	2.76 ^a^	3.03 ^a^	2.91 ^a^	3.27 ^a^	0.3736

Within each row and for each factor, values with the same letter (^a, b, c^) do not differ significantly (*p* < 0.05) when subjected to the one-way analysis of variance (ANOVA) with Tukey’s multiple comparison test.

**Table 5:** Composition of experimental gluten-free bread.

Ingredient (%)	Control	FOCE25%	FOCE50%	FOCE75%	FOCE100%
Corn starch	36.7	36.7	36.7	36.7	36.7
Potato starch	8.9	8.9	8.9	8.9	8.9
Pectin	2.2	2.2	2.2	2.2	2.2
Sugar	2.8	2.8	2.8	2.8	2.8
Salt	0.8	0.8	0.8	0.8	0.8
Oil	1.4	1.4	1.4	1.4	1.4
Fresh yeast	2.8	2.8	2.8	2.8	2.8
FOCE ^1^	-	11.1	22.2	33.3	44.4
Water	44.4	33.3	22.2	11.1	-

^1^ FOCE—Flaxseed oil cake extract.

**Table 6:** Sensory attributes, their definitions, and scale edges used in the quantitative descriptive analysis (QDA).

Attribute	Definition	Scale Edges
Appearance		
beige color	color intensity (color pattern RAL 075 90 10)	Light–dark
pore collocation	visual impression of the arrangement of crumb porous	irregular–regular
pore dimension	visual impression of the size of crumb porous	small–big
Odor		
acid	characteristic of organic acids	none–very intensive
oily	characteristic of sunflower oil	none–very intensive
wheat bread	typical for wheat bread	none–very intensive
sweet	characteristic to sweet baked products	none–very intensive
Texture (manual)		
elasticity	extent to which a product returns to its original length when pushed by a finger	small–big
Texture (in the mouth)		
chewiness	multiplicity of chewing allowing to swallow	low–high
adhesiveness	degree of adhesiveness perceived while chewing the sample 10 times	low–high
moisture	degree of amount of water in the product perceived while chewing the sample 10 times	low–high
Taste		
wheat bread	as the corresponding odor (measured in the mouth)	none–very intensive
acid	basic taste illustrated by citric acid dissolved in water	none–very intensive
sweet	basic taste illustrated by 3% sucrose dissolved in water	none–very intensive
salty	basic taste illustrated by 3% NaCl dissolved in water	none–very intensive
aftertaste	lingering sensation after swallowing the sample	none–very intensive
Overall quality	overall quality contains all attributes and their harmonization	low–high

## Data Availability

Data are contained within the article.

## Sample Availability

Samples are available from the authors.
